# Low positivity rates for HBeAg and HBV DNA in rheumatoid arthritis patients: a case–control study

**DOI:** 10.1186/s12879-022-07536-7

**Published:** 2022-06-24

**Authors:** Yue Jia, Jingjing Zhang, Lingfei Mo, Bomiao Ju, Nan Hu, Yanhua Wang, Pei Wang, Jie Zheng, Lan He, Jing Wang

**Affiliations:** 1 Xi’an Jiaotong University, Xi’an, Shaanxi Province China; 2 Department of Rheumatology and Immunology, the First Affiliated Hospital of Xi’an Jiaotong University, No. 277 Yanta Road (w), Xi’an, 710061 Shaanxi Province China; 3 Clinical Research Center, the First Affiliated Hospital of Xi’an Jiaotong University, Xi’an, Shaanxi Province China

**Keywords:** Rheumatoid arthritis, HBV, Chronic hepatitis B, HBeAg, HBV DNA

## Abstract

**Background:**

The rates of hepatitis B virus (HBV) infection in rheumatoid arthritis (RA) patients are controversial when considering the reported outcomes. It was speculated that HBV infection status was altered after RA, and variations inn HBV infection rates became apparent.

**Methods:**

To compare the positive proportions of hepatitis B e antigen (HBeAg) and HBV DNA, a retrospective case–control study was performed between 27 chronic hepatitis B (CHB) patients with RA and 108 age- and gender-matched CHB patients. In addition, the positivity rates of hepatitis B surface antigen (HBsAg) and hepatitis B core antibody (anti-HBc) were surveyed among the 892 RA patients.

**Results:**

Compared to CHB patients, CHB patients with RA exhibited lower rates of HBeAg positivity (11.1% vs. 35.2%, *P* = 0.003), HBV DNA positivity (37.0% vs. 63.9%, *P* = 0.007) and ALT elevation (11.1% vs. 35.2%, *P* = 0.024). In the 892 RA patients, the prevalence of HBsAg (3.0%) was lower than that reported in the Chinese national data (7.2%), whereas the anti-HBc positivity rate of 44.6% was higher than that of 34.1%.

**Conclusion:**

HBV infection status was altered after suffering from RA. Compared to the matched CHB patients, low positive proportions of HBeAg and HBV DNA were observed for CHB patients with RA.

## Introduction

It was observed that HBV infection affected rheumatoid arthritis (RA), where HBV was considered the suspected trigger for arthritis in genetically susceptible individuals [1]. The rates of positivity for RF and ACPA were as high as 14.4% and 4.1%, respectively, in patients with chronic hepatitis B (CHB) [2]. Hepatitis B core antigen (HBcAg) was found in the synovium of RA patients with CHB, indicating that HBV may be involved in the pathogenesis of local lesions [3]. In RA patients, immune dysregulation and immunosuppressive therapies also influence HBV infection [4, 5].

HBV infection with a high endemicity was reported in various regions of the Asia–Pacific and sub-Saharan Africa [6, 7]. It also affects approximately 10 million people in China [8]. Unfortunately, the HBV infection rates have been reported to be different for RA patients in previous studies. Yilmaz et al. reported a lower HBV infection prevalence in RA patients according to Turkish national data in comparison with the general population [9]. Mahroum et al. performed a case–control study and found that RA patients had a greater proportion of chronic HBV infection than age- and sex-matched controls [10]. Hsu et al. observed that RA patients were characterized by an increased risk of HBV infection when compared with that of the ≥ 18 years-old non-RA cohort [11]. The reasons for these differences were complicated, especially when there were no studies assessing the HBV infection status in RA patients, including HBeAg-positivity, HBV DNA load and ALT level.

Herein, a case–control study was performed to clarify the effect of RA on HBV infection status. The positivity rates of hepatitis B e antigen (HBeAg) were compared between the RA patients with CHB and the age- and gender-matched general CHB patients, in addition to the positivity rates of HBV DNA.

## Methods

### Study design

This was a retrospective case–control study. A total of 27 CHB patients with RA were enrolled from the Department of Rheumatology and Immunology, First Affiliated Hospital of Xi’an Jiaotong University, from January 1st 2016 to December 31st 2019. Inclusion criteria: (i) HBsAg was positive for more than 6 months; (ii) patients fulfilled ACR/EULAR 2010 rheumatoid arthritis classification criteria. The exclusion criteria were serologic human immunodeficiency virus (HIV), hepatitis C (HCV) or hepatitis D virus (HDV) positivity and cirrhosis, liver cancer or fatty liver disease. The diagnosis of cirrhosis was based on a physical examination, biochemical parameters (liver function test, full blood count and prothrombin time) and imageological examination (ultrasonic tests, CT, MR imaging or liver stiffness measurements). A liver biopsy was implemented in cases where the above tests reveal inconclusive results. The fatty liver disease was determined by the evidence of hepatic steatosis, either by imaging or by histology [12]. Arthritis is one of the extrahepatic manifestations in the patients with HBV infection [13]. We can identify RA from arthritis associated with HBV infection by the history of HBV infection, joint deformities, other organ/tissue lesions or deposition in the synovium of circulating immune complexes containing HBsAg-anti-HBs. To exclude the effect of antiviral therapy on HBV infection status, RA patients who accepted antiviral treatment were not included in the matched case–control study. During the corresponding period, age- and gender-matched CHB outpatients were enrolled at a 1:4 ratio from the Department of Infectious Disease. In addition, the positivity rates of HBsAg and anti-HBc were surveyed among the 892 RA patients over the corresponding period. This study was conducted in accordance with the Declaration of Helsinki, the protocol was approved by the Ethics Committee of First Affiliated Hospital of Xi’an Jiaotong University (No. 2017.120), and the patients gave their written informed consent.

The medical records of patients were reviewed, and the data of the following variables were collected: age, sex, diagnosis, duration of disease, HBsAg, HBeAg, anti-HBc, ALT, and HBV DNA load. All the test data of RA patients were collected at the first visit, and the data of CHB patients were collected before their antiviral treatment.

The primary outcome was the comparison of the positivity rates of HBeAg and HBV DNA between the CHB patients with RA and the age- and gender-matched CHB patients. The secondary outcomes were the following: (i) the comparison of the elevated ALT rates between the CHB patients with RA and the CHB patients; (ii) the comparison of the HBeAg titer, HBV DNA load and ALT levels between CHB patients with RA and the CHB patients; (iii) the comparison of HBsAg-positivity rates between the RA patients and Chinese general population (CGP); and (iv) the comparison of anti-HBc-positivity rates between the RA patients and CGP.

### Laboratory methods

The titers of HBsAg, HBeAg and anti-HBc were quantified by Abbott ARCHITECT assays (Abbott Laboratories, Chicago, IL, USA). The lower limit of detection for HBsAg was 0.05 IU/mL, HBeAg was 1 s/co and anti-HBc was 1 s/co. The HBV DNA load was measured by the Roche COBAS AmpliPrep/COBAS TaqMan HBV test (Roche Molecular Systems, California, IL, USA), and the lower limit of detection was 12 IU/mL. Liver function tests were performed with an automated bioanalyzer (Olympus AU5400, Japan).

### Statistical analysis

The analyses were performed by SPSS software 13.0 (SPSS Inc. Chicago, IL, USA). Conditional logistic regression was used to compare the proportions between the two groups. Quantitative data were analyzed with the Shapiro–Wilk test and Levene statistic for normality and homogeneity of variance, respectively. According to the situation, the paired-samples *t*-test or signed rank Wilcoxon test was used to evaluate differences between two groups. A *P* value < 0.05 was considered statistically significant.

## Results

### Basic characteristics of the CHB patients with RA

A total of 27 (3.0%) RA patients were HBsAg-positive and enrolled in this study. The baseline data were collected before disease modifying antirheumatic drugs (DMARDs) treatment in the 26 patients. One patient had methotrexate and hydroxychloroquine treatment before data collection but had discontinued for 11 months. Among the 27 patients, seven patients (25.9%) had a HBV family history, seventeen patients (63.0%) had a HBV infection duration of more than 10 years, and one patient (3.7%) had a RA duration of more than 10 years (Table 1).

**Table 1:** Basic characteristics of the RA patients with HBV infection

Variable	Value
Age, years*	52.0 (28.0–74.0)
Gender, female (%)	19 (70.4)
Duration of RA, years*	3.0 (0.2–18.0)
> 10	1 (3.7)
1–10	19 (70.4)
< 1	7 (25.9)
HBV family history (%)	7 (25.9)
Duration of HBV infection, years	
> 20	11 (40.7)
10–20	6 (22.2)
1–10	2 (7.4)
Unknown	8 (29.6)
ALT, U/L*	15.0 (7.0–476.0)
HBV DNA > 10^2^ IU/mL (%)	10 (37.0)
HBeAg-positive (%)	3 (11.1)

*ALT* alanine transaminase; *HBeAg* hepatitis B e antigen; *HBV* Hepatitis B virus; *RA* rheumatoid arthritis

*The values were expressed as the median (range)

### Low proportions of HBeAg-positive and HBV DNA-positive in patients with CHB and RA

**Fig. 1:**
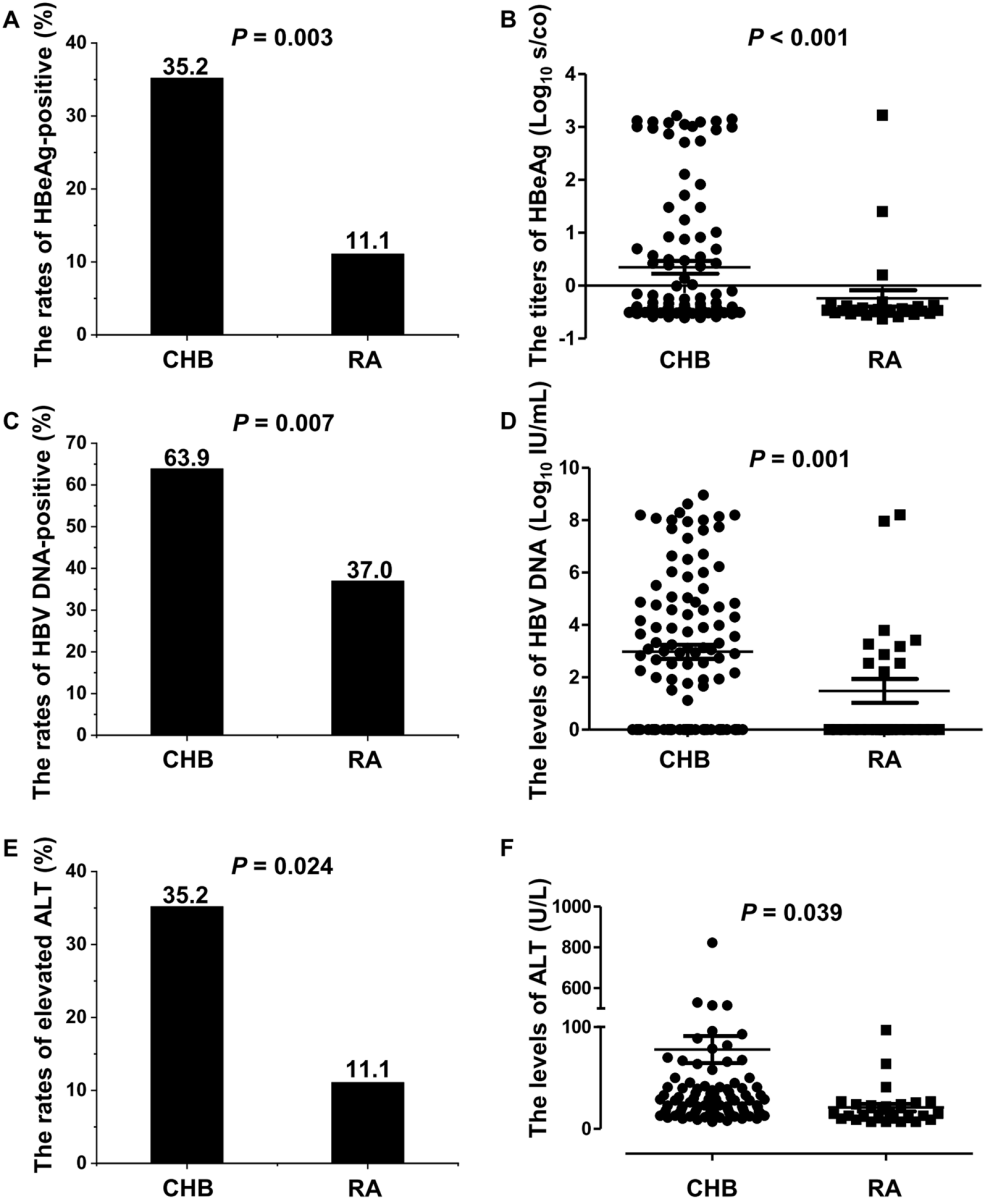
The comparison of HBV infection status between the RA patients and the age- and sex-matched CHB patients: **A** The comparison of HBeAg(+) rates; **B** The comparison of HBeAg titers; **C** The comparison of different HBV DNA gradients rates; **D** The comparison of HBV DNA load; **E** The comparison of elevated ALT rates; **F** The comparison of ALT levels. *ALT* alanine transaminase; *CHB* chronic Hepatitis B; *HBeAg* hepatitis B e antigen; *HBsAg* hepatitis B surface antigen; *HBV* hepatitis B virus; *RA* rheumatoid arthritis

As shown in the methods, 108 age- and gender-matched CHB outpatients were enrolled from the Department of Infectious Disease over the same period in this case–control study. In CHB patients with RA, the proportion of HBeAg(+) patients was 11.1% (3/27), which was much lower than that of the matched CHB patients (11.1% vs. 35.2%; *OR* 0.128; 95% CI 0.033–0.493; *P* = 0.003; Fig. 1A). The titers of HBeAg were lower in the RA patients than in the CHB patients [− 0.5 (− 0.6 to 3.2) vs. − 0.4 (− 0.6 to 3.2), *Z* = − 4.517, *P* < 0.001]. For three HBeAg(+) patients, the titers of HBeAg were 0.2, 1.4 and 3.2 log_10_ s/co, respectively. The corresponding value for the 38 CHB patients was 1.6 (0.02–3.2) log_10_ s/co (Fig. 1B).

The HBV DNA load represents the degree of HBV replication. The positivity rate of HBV DNA in the RA patients was less than that in the matched CHB patients (37.0% vs. 63.9%; *OR* 0.244; 95% CI 0.089–0.674; *P* = 0.007; Fig. 1C). In addition, the HBV DNA load in the RA patients was lower than that in the CHB patients (1.5 ± 2.4 vs. 4.4 ± 3.3 log_10_ IU/mL, *t* = − 3.859, *P* = 0.001, Fig. 1D).

The elevated ALT showed hepatitis activity. Compared to the matched CHB patients, the proportion of elevated ALT (> 40 U/L) was significantly lower in the RA patients (11.1% vs. 35.2%; *OR* 0.233; 95% CI 0.066–0.824; *P* = 0.024; Fig. 1E), together with the level of ALT [15.0 (7.0–97.0) vs. 22.0 (10.0–476.0), *Z* = − 2.066, *P* = 0.039, Fig. 1F].

### Low prevalence of HBsAg and high prevalence of anti-HBc in RA patients

**Fig. 2:**
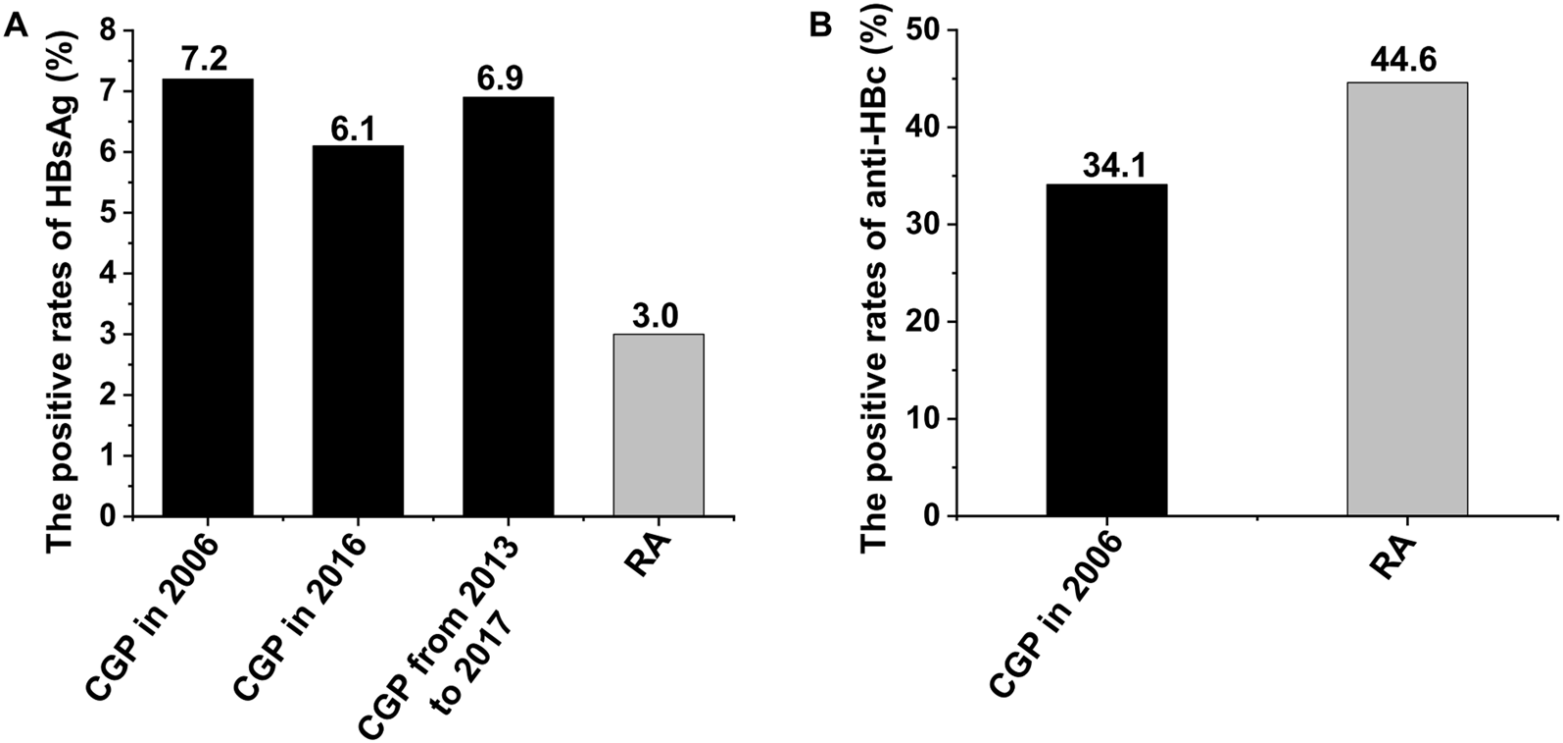
The comparison of HBsAg(+) rate and anti-HBc(+) rates between the RA patients and the Chinese general population: **A** The comparison of HBsAg(+) rates; **B** The comparison of anti-HBc(+) rates. *The CGP data: CGP in 2006 [8], CGP in 2016 [14] and CGP from 2013 to 2017 [15]. *anti-HBc* hepatitis B core antibody; *CGP* Chinese general population; *HBsAg* hepatitis B surface antigen; *RA* rheumatoid arthritis

With respect to the rate of HBsAg-positivity, the second Chinese National Hepatitis Seroepidemiological Survey demonstrated that the rate was 7.2% in 2006 (Fig. 1A) [8]. Then, the Polaris Observatory Collaborators developed models for 120 countries, and estimated that the prevalence of HBsAg in China in 2016 was 6.1% (5.5–6.9%) [14]. Based on the 27 included studies, Wang et al. estimated prevalence of 6.89% (5.84–7.95%) for HBV infection in the general population of China from 2013 to 2017 [15]. In the present work, 3.0% of RA patients (27/892) were HBsAg-positive. This is lower than the above reported data (Fig. 2A).

Anti-HBc positivity mostly occurs in chronic HBV infection or resolved infection [16]. The anti-HBc(+) rate was 44.6% (398/892) in RA patients, higher than the data of the Chinese National Hepatitis Seroepidemiological Survey (44.6% vs. 34.1%, Fig. 2B).

## Discussion

For RA patients, the reported difference in HBV infection rates may be associated with the alteration of HBV infection status after suffering from RA. To elucidate this issue, the current case–control study was performed. Compared to the age- and gender-matched general CHB patients, low proportions of positivity for HBeAg and HBV DNA were observed for the CHB patients with RA.

HBeAg positivity often represents a high replicative phase of chronic HBV infection, and HBeAg loss is considered partial immune control of chronic HBV infection [17]. In this study, CHB patients with RA exhibited lower positivity rates of HBeAg and titers of HBeAg than matched CHB patients. HBV DNA directly indicates HBV replication. The positivity rate of HBV DNA in the CHB patients with RA was less than that of the matched CHB patients, as was the HBV DNA load. It demonstrated that CHB patients with RA had a higher probability of HBeAg seroconversion and HBV DNA load decline, which may be associated with immune control after suffering from RA.

Immune dysregulation is the a characteristic of RA. It plays a complicated role in HBV infection for abnormal innate and adaptive immune activation in RA patients. First, type I interferons (IFNs) play a critical role in defending against HBV, and the type I interferon signature is detectable in the peripheral blood of RA patients [18]. Second, CD8+ T cells are capable of controlling HBV infection and eliminating HBV infected cells [19]. For RA patients, CD8+ T cells are abundant and associated with disease activity, due to pro-inflammatory cytokine production [20] and self-antigens responses upon cross-presentation [21]. Third, the humoral immune response has a protective role against pathogens [22]. Abnormalities in B cells not only participate in the pathogenesis of RA [23] (including the production of autoantibodies, presentation of autoantigens and secretion of proinflammatory cytokines) [24], but also affect HBV elimination.

The elevated ALT is an important characteristic of immune clearance [25], and inactive HBsAg carrying status can be obtained after immune clearance. Compared to the matched CHB patients, low proportion of ALT > 40 U/L and low ALT levels were found for the CHB patients with RA. This suggested that CHB patients with RA were more prone to obtain immune control for HBV after immune clearance.

HBsAg positivity is a definite HBV infection marker. In the present work, low HBsAg(+) rate of 3.0% was found in RA patients, which was consistent with a previous study [9]. However, the prevalence of HBsAg-positive was lower than other studies [10, 11, 26], which may be associated with the different ages, regions of patients, sample sizes and methods of HBsAg testing. Our data indicated that RA patients exhibited a low HBsAg(+) rate according to the second Chinese National Hepatitis Seroepidemiological Survey [8] and the estimated prevalence of HBsAg in China [14, 15]. Hepatitis B core antigen (HBcAg) is an inner nucleocapsid component, and the production of anti-HBc is induced by a cellular and humoral immune response to HBcAg during natural HBV infection. Anti-HBc positivity mostly occurs in chronic HBV infection or resolved infection [16]. We found that the positivity rate of anti-HBc was 44.6% in the RA patients, higher than the rate in the Chinese general population from the China national data [8]. Consistent with the previous studies [10, 11], RA patients may have a higher risk of HBV infection than the general population, due to receiving disease modifying antirheumatic drugs and complicated immunity related to the disease itself [4, 5]. During the natural history of HBV, HBsAg seroclearance can emerge in 0.5–1.0% patients per year after immune clearance phase [27]. It was expected that a low positivity rate of HBsAg would indicate that HBsAg seroclearance was more common in RA patients. The susceptible ages were different for RA and CHB patients. Mother-to-infant transmission was the main route of HBV infection in China. HBV infects early in life, and confers a high risk of chronicity [28]. In contrast, RA occurs much more frequently in elderly women. Hence, RA was supposed to be later than HBV infection for most patients. We speculated that HBV infection status altered after suffering from RA. Among the 27 patients in this study, 62.9% had a HBV duration of more than 10 years, and 96.3% had aRA duration of less than 10 years. This indicates that the majority suffered from RA after HBV infection. After suffering from RA, HBV DNA declines, and HBeAg and even HBsAg lost.

Here, some limitations of the study were as follows: the numbers of patients were limited, and a prospective cohort study with large sample size is necessary to evaluate the difference in the natural history of chronic HBV infection between RA and general CHB patients.

In conclusion, HBV infection status was altered after suffering from RA. Compared to the matched CHB patients, variations were significant, including low positive proportions of HBeAg and HBV DNA, due to immune dysregulation of RA patients.

## Data Availability

The datasets generated and/or analyzed during the current study are not publicly available (since the host institution for the research to makes data available only upon request) but are available from the corresponding author on reasonable request.
